# Influence of Maternal and Child Lifestyle-Related Characteristics on the Socioeconomic Inequality in Overweight and Obesity among 5-year-old Children; The “Be Active, Eat Right” Study

**DOI:** 10.3390/ijerph10062336

**Published:** 2013-06-06

**Authors:** Lydian Veldhuis, Ineke Vogel, Lenie van Rossem, Carry M. Renders, Remy A. HiraSing, Johan P. Mackenbach, Hein Raat

**Affiliations:** 1 Department of Public Health, Erasmus MC-University Medical Centre Rotterdam, Dr Molewaterplein 50, Rotterdam 3000 CA, The Netherlands; E-Mails: i.vogel@erasmusmc.nl (I.V.); l.vanrossem@umcutrecht.nl (L.R.); j.mackenbach@erasmusmc.nl (J.P.M.); h.raat@erasmusmc.nl (H.R.); 2 Institute of Health Sciences, Faculty of Earth and Life Sciences & EMGO Institute for Health and Care Research, VU University Amsterdam, Amsterdam 1081 HV, The Netherlands; E-Mail: carry.renders@vu.nl; 3 Department of Public and Occupational Health, EMGO Institute for Health and Care Research, VU University Medical Centre, Amsterdam 1007 MB, the Netherlands; E-Mail: ra.hirasing@vumc.nl

**Keywords:** preschool child, overweight, obesity, social class, lifestyle

## Abstract

It is unclear whether the socioeconomic inequality in prevalence of overweight and obesity is already present among very young children. This study investigates the association between overweight and socioeconomic status (SES, with maternal educational level as an indicator of SES) among 5-year-old children. This cross-sectional study uses baseline data from 5-year-olds of Dutch ethnicity (n = 5,582) and their mothers collected for the “Be active, eat right” study. Compared to children of mothers with the highest educational level, for children of mothers with the lowest educational level the odds ratio (adjusted for demographic characteristics) for having overweight was 2.10 (95% confidence interval: 1.57–2.82), and for having obesity was 4.18 (95% confidence interval: 2.32–7.55). Addition of maternal and child lifestyle-related characteristics decreased the odds ratios for overweight and obesity by 26.4% and 42.1%, respectively. The results show that an inverse SES-overweight/obesity association is already present at elementary school entry, and that watching TV by mother and child, the child consuming breakfast and, especially maternal weight status, are contributing factors in this association. These results should be taken into account when developing policies to reduce inequalities in (childhood) health.

## 1. Introduction

Childhood overweight and obesity have increased at a dramatic rate worldwide and are a major burden on public health [1,2]. Compared to higher socioeconomic status (SES) groups, subgroups in society with a lower SES are at increased risk for having overweight or obesity [1] and these differences in prevalence of overweight across SES groups may be established early in life [3,4,5]. In the last decades, studies investigating the association between SES and overweight among schoolchildren indeed found predominately inverse associations [4,6,7,8]. Results of studies among younger children (aged < 6 years) are however less consistent; some studies did find an association during early childhood [8,9,10,11,12], while others did not [13,14,15]. Differences in the prevalence of childhood overweight across SES groups are likely to be explained by differences in characteristics of the children and their parents related to material circumstances, behavior and/or knowledge, all of which influence energy balance [6,9]. Only few studies evaluated to what extent parental overweight and lifestyle-related behaviors of the child, *i.e.*, playing outside, sedentary behavior, consuming breakfast and drinking sweet beverages, contribute to the association between SES and early childhood overweight [6,7]; these lifestyle-related behaviors have been shown to be associated with both childhood overweight and indicators of SES [16,17,18,19,20]. Especially having an overweight parent is an important factor likely to influence the SES-overweight association among children, *i.e.*, parental overweight reflects a combination of inherited genes and shared environment, and children are likely to learn behaviors related to energy intake/expenditure from their parents [5,6,9,21,22]. Understanding the influence of SES on patterns of eating and physical activity that lead to early childhood overweight and obesity is critical for the development of effective prevention programs. 

In summary, more research is needed to establish whether socioeconomic inequality in the prevalence of overweight and obesity is already present in early childhood, also with regard to a timely start of overweight prevention programs. Therefore this study evaluates whether the educational level of the parent, as an indicator of SES, is associated with overweight/obesity among a large sample of 5-year-old children. Also investigated is the extent to which a potential association can be explained by lifestyle-related characteristics of the parent and child.

## 2. Methods

### 2.1. Design and Study Population

The present cross-sectional study is embedded in the “Be active, eat right” study, which aims to assess the effects of an overweight prevention protocol among children at elementary school entry, throughout the Netherlands, as detailed elsewhere [23]. The Medical Ethics Committee of Erasmus MC University Medical Centre Rotterdam approved the study protocol.

Of the 37 municipal health services in the Netherlands, an opportunity sample of nine municipal health services agreed to participate in the “Be active, eat right” study. A total of 13,638 parents of 5-year-olds were invited by mail for a well-child visit at one of these nine municipal health services. These parents were also invited to participate in the “Be active, eat right” study and 64.4% provided written informed consent to participate in the study. Baseline data were collected during the 2007–2008 school year and were used for the present study.

Parental educational level is suggested to be an indicator of SES [24]. Besides material resources, parental educational level can also reflect a range of non-economic social characteristics with important health effects, such as health-related knowledge [24]. Parental educational level is likely to be a relatively stable indicator compared to, for example, parental income. It is also suggested that, especially the educational level of the mother, has a considerable influence on the development of children [6,25].

The present study used data of the children for whom the mother completed the questionnaire (n = 7,682). In addition, we included an ethnically homogenous group as the association between SES and early childhood overweight may differ between ethnic subgroups [7,24,26]. Children with a Dutch ethnicity comprised the largest ethnic subgroup and were selected for analysis in the present study (n = 6,641). A child was considered to be of non-Dutch ethnicity when at least one of the parents was born abroad, as defined by Statistics Netherlands [27].

Children were excluded from the analysis when data were missing on height or weight of the child (n = 20), sex or age of the child (n = 6), lifestyle-related characteristics of the child (consuming breakfast, drinking sweet beverages, playing outside and watching television; TV) (n = 685), educational level of the mother (n = 26), age of the mother (n = 6), single parenting or employment status of the mother (n = 53), watching TV by the mother (n = 166), and height or weight of the mother (n = 97). After exclusion for any of these reasons, a study population of n = 5,582 children remained for analysis.

### 2.2. Educational Level of the Mother

Maternal educational level was assessed by a questionnaire completed by the mothers. Educational level was recoded in three categories according to the Dutch standard classification as defined by Statistics Netherlands [28], allowing meaningful comparison between subgroups of different educational level: high level (academic higher education/university education, higher professional education), mid level (pre-university education, senior general secondary education, and senior secondary vocational education), and low level (preparatory secondary vocational education, lower secondary vocational education, primary education, and no education).

### 2.3. Weight Status of the Child

Body weight and height of the children were measured by trained healthcare professionals of the municipal health services using standardized methods as described in a protocol [17]. Body weight was measured to the nearest 0.1 kg and height to the nearest 0.1 cm. Body mass index (BMI) was calculated by dividing weight (in kilograms) by height (in meters) squared. The weight status of the children was assessed according to the age-specific and sex-specific cut-off points for BMI as published by the International Obesity Task Force [29]. When a child’s BMI value was the same as or higher than the lower-bound cut-off point for overweight or obesity for the child’s age and sex, the child was classified as having overweight or obesity.

### 2.4. Demographic and Lifestyle-Related Characteristics of the Mother and Child

Information on demographic and lifestyle-related characteristics of the mother and child were obtained via the questionnaire completed by the mothers. The categories are indicated below in parentheses. Of the maternal characteristics, we considered age, single parenting (two-parent families, single-parent family or otherwise specified), and employment status (employed full-time or part-time, not employed) to be potential confounders in the association between educational level of the mother and weight status of her child. Maternal weight status (no overweight, overweight, obesity) and watching TV (≤2 h/day, >2 h/day) by the mother were considered to be potential mediating characteristics. Self-reported height and weight of the mothers were used to calculate the BMI of the mothers. Mothers were classified as overweight when the BMI value was ≥25–30 kg/m^2^ and were classified obese when the BMI value was ≥30 kg/m^2^, as defined by the World Health Organization [2]. For the children, we considered age and sex to be potential confounders, and the lifestyle-related characteristics consuming breakfast (daily, <7 days/week), drinking sweet beverages (*i.e.*, lemonade, soda, carbonated soda, fruit juice, sugar sweetened dairy products, *etc.*) (≤2 glasses/day, >2 glasses/day), playing outside (≥1 h/day, <1 h/day) and watching TV (≤2 h/day, >2 h/day) to be potential mediators in the association between educational level of the mother and weight status of the child. The categories used for the behaviors are based on established international recommendations [17,18,19,30,31,32].

### 2.5. Statistical Analyses

Mean and frequency differences of the characteristics of the mothers and children, across maternal educational levels, were examined using analyses of variance (ANOVA) and Chi-square statistics. Multinomial logistic regression analyses were used to test the association between maternal educational level and overweight and obesity of the children. Odds ratios (ORs) and 95% confidence intervals (CI) were obtained for each educational level and compared with the reference category (highest educational level).

The basic model investigated the association between maternal educational level, and overweight and obesity of the children. In the association with children’s overweight (obesity included), there were no interactions between the maternal and child demographic and lifestyle-related characteristics, and maternal educational level. To test the influence of the characteristics on the association, the characteristics were added to the basic model one at a time. For each adjustment, the percentage change in OR was calculated for the educational levels ([OR_basic model+characteristic_ − OR_basic model_]/[OR_basic model_ − 1] × 100) [33]. Subsequently, the association between maternal educational level and children’s overweight and obesity was analyzed, with adjustment for the relevant characteristics. A characteristic was considered relevant if the percentage change in ORs was >5% for an educational level subgroup [33]. This approach was applied to reduce the final number of variables included in the model. First, in Model 1, we adjusted the association for the relevant confounding characteristics of the mother and child. Second, in Model 2, we also adjusted the association for the relevant mediating characteristics of the mother. Finally, in Model 3, we also added the mediating characteristics of the child. Statistical analyses were performed with PASW Statistics 17 for Windows (SPSS Inc, Chicago, IL, USA).

## 3. Results

Compared to the mothers/children with missing data (n = 1,059), the population analyzed (n = 5,582) included significantly more mothers with a high educational level (*p* < 0.001), more mothers that were employed (*p* < 0.001), more mothers that watched TV ≤2 h/day (*p* < 0.05), and more children who consumed breakfast daily (*p* < 0.05) and watched TV ≤2 h/day (*p* < 0.001). There was no significant difference in weight status of the child (*p* = 0.41) or maternal weight status (*p* = 0.14) between the subgroup with missing data compared to the study population (data not shown).

Of all included mothers, mean age was 36.5 (SD 4.1) years, 22.1% had overweight, 6.8% had obesity, and 18.9% were in the group with the lowest educational level. Of the children, 51% were boys, mean age was 5.7 (SD 0.4) years, 6.9% had overweight, and 1.5% had obesity. All demographic and lifestyle-related characteristics of the mother and the child (except for single parenting and child’s sex) were significantly associated with maternal educational level (Table 1).

**Table 1:** Characteristics of the total study population (n = 5,582), and by educational level of the mother.

	Frequency in the study population (%) (unless otherwise specified)				*p-*value ^a^
	Total	Educational level of the mother ^b^			
		High (n = 1,933)	Mid (n = 2,596)	Low (n = 1,053)	
*Characteristics of the mother:*					
Mean age, years (SD)	36.5 (4.1)	37.5 (3.7)	36.1 (4.1)	36.0 (4.7)	<0.001
Mean height, cm (SD)	170.7 (6.2)	171.1 (5.9)	170.6 (6.3)	170.1 (6.2)	<0.001
Mean weight, kg (SD)	69.5 (12.3)	67.9 (11.2)	69.8 (12.5)	71.8 (13.4)	<0.001
Mean BMI (SD)	23.9 (4.0)	23.2 (3.6)	24.0 (4.1)	24.8 (4.4)	<0.001
Overweight ^c^	1,234 (22.1)	326 (16.9)	608 (23.4)	300 (28.5)	<0.001
Obesity ^c^	379 (6.8)	84 (4.3)	186 (7.2)	109 (10.4)	
Single parent	330 (5.9)	114 (5.9)	141 (5.4)	75 (7.1)	0.15
Not employed	1,424 (25.5)	326 (16.9)	667 (25.7)	431 (40.9)	<0.001
Watches TV > 2 h/day	2,537 (45.4)	570 (29.5)	1,315 (50.7)	652 (61.9)	<0.001
*Characteristics of the child*					
Boy	2,824 (50.6)	976 (50.5)	1,319 (50.8)	529 (50.2)	0.95
Mean age, years (SD)	5.7 (0.4)	5.7 (0.4)	5.7 (0.4)	5.8 (0.4)	<0.001
Mean height, cm (SD)	117.7 (5.5)	117.3 (5.4)	117.8 (5.6)	118.1 (5.6)	<0.001
Mean weight, kg (SD)	21.5 (3.2)	21.1 (2.9)	21.5 (3.3)	22.0 (3.5)	<0.001
Mean BMI (SD)	15.4 (1.5)	15.3 (1.3)	15.5 (1.5)	15.7 (1.6)	<0.001
Overweight ^d^	386 (6.9)	95 (4.9)	188 (7.2)	103 (9.8)	<0.001
Obesity ^d^	84 (1.5)	17 (0.9)	32 (1.2)	35 (3.3)	
Consuming breakfast < 7 day/week	295 (5.3)	36 (1.9)	137 (5.3)	122 (11.6)	<0.001
Drinking sweet beverages > 2 glasses/day	3,619 (64.8)	1,124 (58.1)	1,744 (67.2)	751 (71.3)	<0.001
Playing outside < 1 h/day	339 (6.1)	174 (9.0)	133 (5.1)	32 (3.0)	<0.001
Watching TV > 2 h/day	914 (16.4)	169 (8.7)	467 (18.0)	278 (26.4)	<0.001

BMI = body mass index; SD = standard deviation. ^a^ *p*-values for differences in characteristics across maternal educational levels; ANOVA’s were used for continuous variables and Chi-square statistics for dichotomous variables. ^b^ High educational level = academic higher education (university education), higher professional education; mid educational level = pre-university education, senior general secondary education, and senior secondary vocational education; low educational level = preparatory secondary vocational education, lower secondary vocational education, primary education, and no education. ^c^ Overweight = BMI 25–30 (kg/m^2^); obesity = BMI ≥ 30 (kg/m^2^) [2]. ^d^ According to age and sex-specific cut-off points for BMI as published by the International Obesity Task Force [29].

In the basic model the association between maternal educational level and overweight and obesity of the children was investigated (Table 2, basic model). The characteristics that changed the ORs by >5% were: maternal age, maternal weight status, watching TV by the mother and the child, and the child consuming breakfast (Table 2).

**Table 2:** Multinomial logistic regression analyses for the association between maternal educational level and children’s overweight and obesity, and change in ORs after adjustment for demographic and lifestyle-related characteristics of the mother and child (n = 5,582).

	High (ref) OR	Mid OR (95% CI)	Change 1 ^a^	Low OR (95% CI)	Change 2 ^a^
**Overweight (n = 386)**					
Educational level of the mother ^b^ (basic model)	1.00	1.52 (1.18–1.96)		2.16 (1.62–2.88)	
*Characteristics of the mother*					
Basic model + age	**1.00**	**1.47 (1.14–1.90)**	**−9.6%**	**2.10 (1.57–2.82)**	**−5.2%**
Basic model + single parenting	1.00	1.52 (1.18–1.96)	0%	2.15 (1.61–2.88)	−0.9%
Basic model + employment status	1.00	1.53 (1.19–1.97)	1.9%	2.21 (1.64–2.97)	+4.3%
Basic model + weight status ^c^	**1.00**	**1.42 (1.10–1.83)**	**−19.2%**	**1.91 (1.42–2.56)**	**−21.6%**
Basic model + watching TV	**1.00**	**1.49 (1.15–1.93)**	**−5.8% **	**2.10 (1.56–2.83)**	**−5.2%**
*Characteristics of the child*					
Basic model + sex	1.00	1.52 (1.18–1.97)	0%	2.17 (1.62–2.90)	+0.9%
Basic model + age	1.00	1.51 (1.17–1.95)	−1.9%	2.15 (1.61–2.88)	−0.9%
Basic model + consuming breakfast	**1.00**	**1.50 (1.16–1.94)**	**−3.8%**	**2.09 (1.56–2.81)**	**−6.0%**
Basic model + drinking sweet beverages	1.00	1.50 (1.16–1.94)	−3.8%	2.12 (1.59–2.84)	−3.4%
Basic model + playing outside	1.00	1.52 (1.18–1.96)	0%	2.17 (1.62–2.90)	+0.9%
Basic model + watching TV	**1.00**	**1.51 (1.17–1.95)**	**−1.9%**	**2.14 (1.60–2.87)**	**−1.7%**
**Obesity (n = 84)**					
Educational level of the mother ^b^ (basic model)	1.00	1.44 (0.80–2.61)		4.10 (2.28–7.35)	
*Characteristics of the mother*					
Basic model + age	**1.00**	1.49 (0.82–2.70)	+11.4%	**4.18 (2.32–7.55)**	**+2.6%**
Basic model + single parenting	1.00	1.46 (0.81–2.63)	+4.5%	4.04 (2.25–7.25)	−1.9%
Basic model + employment status	1.00	1.43 (0.79–2.59)	−2.3%	4.01 (2.21–7.29)	−2.9%
Basic model + weight status ^c^	**1.00**	1.29 (0.71–2.34)	−34.1%	**3.35 (1.85–6.07)**	**−24.2%**
Basic model + watching TV	**1.00**	1.30 (0.71–2.37)	−31.8%	**3.50 (1.92–6.40)**	**−19.4%**
*Characteristics of the child*					
Basic model + sex	1.00	1.45 (0.80–2.61)	2.3%	4.11 (2.29–7.38)	+0.3%
Basic model + age	1.00	1.44 (0.79–2.59)	0%	4.05 (2.25–7.27)	−1.6%
Basic model + consuming breakfast	**1.00**	1.43 (0.79–2.59)	−2.3%	**4.02 (2.22–7.26)**	**−2.6%**
Basic model + drinking sweet beverages	1.00	1.42 (0.79–2.57)	−4.5%	4.01 (2.23–7.23)	−2.9%
Basic model + playing outside	1.00	1.43 (0.79–2.58)	−2.3%	4.02 (2.24–7.24)	−2.6%
Basic model + watching TV	**1.00**	1.35 (0.75–2.45)	−20.5%	**3.63 (2.00–6.59)**	**−15.2%**

OR = odds ratio; CI = confidence interval. Model Fitting Information: basic model χ^2^ (4) = 52.37, *p* < 0.001. Results of Likelihood Ratio Tests: age mother χ^2^ (6) = 6.13, *p* = 0.41; single parenting χ^2^ (2) = 11.19, *p* < 0.05; employment status mother χ^2^ (2) = 0.75, *p* = 0.69; weight status mother χ^2^ (4) = 59.57, *p* < 0.001; watching TV by the mother χ^2^ (4) = 4.86, *p* = 0.09; sex child χ^2^ (2) = 37.98, *p* < 0.001; age child χ^2^ (6) = 6.85, *p* = 0.34; consuming breakfast child χ^2^ (2) = 2.13, *p* = 0.35; drinking sweet beverages child χ^2^ (2) = 1.73, *p* = 0.42; playing outside child χ^2^ (2) = 0.56, *p* = 0.75; watching TV child χ^2^ (2) = 5.32, *p* = 0.07. In these analyses, age of the mother and the child were included as categorical variables to reduce the number of cells with zero frequencies. ^a^ Change 1 and change 2 represent the change in OR relative to the basic model for mid and low education, respectively, after adjustment for lifestyle/demographic characteristics ([OR_basic model+characteristic_ − OR_basic model_]/[OR_basic model_ − 1] × 100). The changes in ORs >5% are indicated in bold numbers. ^b^ High educational level = academic higher education (university education), higher professional education; mid educational level = pre-university education, senior general secondary education, and senior secondary vocational education; low educational level = preparatory secondary vocational education, lower secondary vocational education, primary education, and no education.^c^ Overweight = BMI 25–30 (kg/m^2^); obesity = BMI ≥ 30 (kg/m^2^) [2].

In the model with adjustment for the confounding characteristic maternal age, compared to children in the subgroup with a mother with the highest educational level, the OR for having overweight for children in the subgroup with a mother with a mid educational level was 1.47 (95% CI: 1.14–1.90), and for children in the subgroup with a mother with the lowest educational level the OR was 2.10 (95% CI: 1.57–2.82). For children in the subgroup with a mother with the lowest educational level, the OR for having obesity was 4.18 (95% CI: 2.32–7.55) (Table 3, Model 1). Compared to Model 1, addition of the mediating characteristics of the mother (weight status and watching TV) and the child (watching TV and consuming breakfast) to the model resulted in a total decrease in the ORs of 21.3% to 42.1% (Table 3, Model 2 and Model 3). For children with a mother with a mid educational level the OR for having overweight in the final model was 1.36 (95% CI: 1.05–1.77), and for children with a mother with the lowest educational level the OR was 1.81 (95% CI: 1.33–2.46). In the final model, for children in the subgroup with a mother with the lowest educational level, the OR for having obesity was 2.84 (95% CI: 1.52–5.29) (Table 3, Model 3).

**Table 3:** Multinomial logistic regression analyses for the association between maternal educational level and children’s weight status (n = 5,582).

	Model 1	Model 2	Change 1 ^a^	Model 3	Change 2 ^a^
	OR (95% CI)	OR (95% CI)		OR (95% CI)	
**Overweight (n = 386)**					
*Educational level of the mother ^b^*					
High	1.00	1.00		1.00	
Mid	1.47 (1.14–1.90)	1.37 (1.06–1.78)	–21.3%	1.36 (1.05–1.77)	–23.4%
Low	2.10 (1.57–2.82)	1.85 (1.37–2.51)	–22.7%	1.81 (1.33–2.46)	–26.4%
**Obesity (n = 84)**					
*Educational level of the mother ^b^*					
High	1.00	1.00		1.00	
Mid	1.49 (0.82–2.70)	1.22 (0.67–2.24)	–55.1%	1.19 (0.64–2.18)	–61.2%
Low	4.18 (2.32–7.55)	3.03 (1.64–5.60)	–36.2%	2.84 (1.52–5.29)	–42.1%

OR = odds ratio; CI = confidence interval. Model 1: educational level of the mother, adjusted for relevant confounding characteristics (maternal age). Model 2: Model 1 + relevant mediating characteristics of the mother (weight status, watching TV). Model 3: Model 2 + relevant mediating characteristics of the child (consuming breakfast, watching TV). Reference category is the subgroup of children without overweight. In these analyses, age of the mother and the child were included as categorical variables to reduce the number of cells with zero frequencies. ^a^ Change 1 and change 2 represent the change in OR relative to Model 1 for mid and low education, after adjustment for the mediating characteristics of the mother (Model 2) and the child (Model 3) ([OR_Model 2/3_ − OR_Model 1_]/[OR_Model 1_ − 1] × 100). ^b^ High educational level = academic higher education (university education), higher professional education; mid educational level = pre-university education, senior general secondary education, and senior secondary vocational education; low educational level = preparatory secondary vocational education, lower secondary vocational education, primary education, and no education.

## 4. Discussion

This study shows an inverse association between maternal educational level as an indicator of SES, and overweight and obesity among children at age 5 years. Adjustment for the mediating characteristics maternal weight status and watching TV by the mother decreased the odds for having overweight for a child in the subgroup with the lowest SES by 22.7%; for obesity, this decrease was 36.2%. Consuming breakfast and watching TV by the child, decreased these odds further by respectively 3.7% and 5.9%. 

For the present study a large sample of young children throughout the Netherlands was included (n = 5,582). However, this was an opportunity sample of nine out of 37 municipal health services that were able and willing to participate in the “Be active, eat right” study. Because of the small age range, our results are specific to the 5-year-old age group. In addition, use of an ethnically homogenous group allowed to avoid the effect of ethnicity when evaluating the effect of educational level on overweight and obesity. The prevalence of overweight and obesity in our study population was 6.9% and 1.5%, respectively. In comparison, the prevalence rates for Dutch 5-year-olds in 2009 presented by a nationwide study were approximately 15.5% for overweight and 2.7% obesity [34]. There appeared to be some selection towards a study population with a higher SES and a somewhat healthier lifestyle, and the prevalence of overweight and obesity is probably underestimated in our study. Therefore, results should be generalized with caution. However, a clear inverse association was found between maternal educational level and children’s risk for having overweight/obesity in our study population and (although we cannot confirm this) we think it unlikely that this association differs in the subgroup with missing data, or in the source population of Dutch 5-year-olds. Further, additional analysis after inclusion of the subgroup of children with missing values on lifestyle-related characteristics revealed that the results were similar to the included children (data not shown). Based on this, we assume that selection bias may not be a major threat to the validity of our results.

There are also other methodological considerations that need to be addressed. Limitations of this study are the use of cross-sectional data and the use of self-reported data for the characteristics of the mother and child. Maternal educational level was recoded in three categories, allowing meaningful comparisons between subgroups of different educational level. However, the subgroup of mothers categorized as having a low educational level is fairly heterogeneous; also mothers that reported no education at all were included in this subgroup (0.2% of the mothers in the total study population). Further, no information was available in the present study on, for example, physical activity of the parents, characteristics of the neighborhood environment (perceived safety, availability of parks, playgrounds, bike paths, *etc.*) [7], prenatal, perinatal and postnatal factors (such as maternal smoking during pregnancy, birth weight, and receiving breastfeeding) [14]. With regard to watching TV by the child and the mother, in this study, we asked in the questionnaire how much time was spent watching TV; and not for how long the TV was turned on during the day. However, for future studies we recommend to distinguish between watching TV as a primary activity, and having the TV turned on in the background in combination with other activities. Further, parents might have given socially desirable answers, although anonymity was assured. The height/weight of the children, on the other hand, was measured by trained healthcare professionals of the municipal health services.

A literature review (including 45 cross-sectional studies performed 1989–2005) concluded that school-aged children whose parents (particularly mothers) have a lower level of education were at higher than average risk to have overweight [6]. Relatively recent studies among 3-year-olds [13,14] and 4-year-olds [15] found no association between SES and childhood overweight. The results of these previous studies, together with our results, suggest that (currently) differences in the prevalence of childhood overweight across SES-levels appear at the time of elementary school entry. Earlier studies including children at the age of about 5 years confirm this trend [8,9,10,11,12].

Our study adds to the existing knowledge by demonstrating to what extent the inverse association between SES and early childhood overweight and obesity can be explained by lifestyle-related characteristics of the mother and child, including maternal overweight. Maternal weight status is a complex factor that can influence the SES-overweight/obesity association among children, as it represents both shared genes and lifestyle [5,6,9,21,22]. Maternal weight status appeared to be the most important explanatory factor in the present study, although the associations between SES and overweight/obesity among the children remained significant after adding this to the model. Lifestyle-related characteristics of the mother and the child further explained the increased risk for overweight/obesity for the lowest SES subgroup, but the SES-overweight/obesity association remained significant. Thus, the factors analyzed in the present study did not totally explain the SES-overweight/obesity association. Other factors such as characteristics of the neighborhood environment (perceived safety, availability of parks, playgrounds, bike paths, *etc.*) [7], prenatal, perinatal and postnatal factors (such as maternal smoking during pregnancy, birth weight, and receiving breastfeeding) [14] have been suggested in the literature as potential explanatory factors of the SES-overweight/obesity association, however, these factors were not available in the present study. We recommend future studies to include environmental factors, prenatal, perinatal and postnatal factors, parenting style and parenting practices, and specific measures of diet, sedentary and physical activity behaviors over time, to further explain differences in prevalence of early childhood overweight among subgroups of different SES.

## 5. Conclusions

In conclusion, this study shows that there is already a difference between SES groups in the presence of overweight and obesity at the start of elementary school. Compared to children of mothers with the highest educational level, the children of mothers with a lower educational level are at increased risk to have overweight and, in particular, to have obesity. This higher risk for the lower SES groups is explained by maternal and child lifestyle-related characteristics for >25% and >40%, respectively. Because differences in childhood overweight across SES subgroups may increase over time, which may contribute to increasing health inequalities [5,13,22], it is important to start interventions to prevent overweight and obesity early in life. When developing overweight prevention programs for young children, the differences in risk should be taken into account and attention should be paid to the specific influence of maternal weight status and watching TV, as these factors appear to substantially contribute to the differences in risk for gaining overweight or obesity in children across SES subgroups.
